# Protocol to discover machine-readable entities of the ecosystem management actions taxonomy

**DOI:** 10.1016/j.xpro.2024.103125

**Published:** 2024-06-13

**Authors:** Timothy C. Haas

**Affiliations:** 1 Sheldon B. Lubar College of Business, University of Wisconsin, Milwaukee, WI, USA

**Keywords:** Bioinformatics, Computer sciences, Environmental sciences

## Abstract

The ecosystem management actions taxonomy (EMAT) consists of actions taken by humans and wildlife that affect an ecosystem. Here, I present a protocol for discovering machine-readable entities of the EMAT. I describe steps for acquiring stories from online locations, collecting them into a story file, and processing them through a software package to extract those actions that match EMAT taxa. I then detail procedures for using the story file to learn new EMAT taxa.

## Highlights

• Protocol for the collection of data on the fundamental variables of conservation science
• Story acquisition from online locations, story file collection, and processing
• Steps for discovering new EMAT taxa using a software-assisted technique

## Abstract (editor highlights)

Publisher’s note: Undertaking any experimental protocol requires adherence to local institutional guidelines for laboratory safety and ethics.

## Graphical abstract

**Figure:**
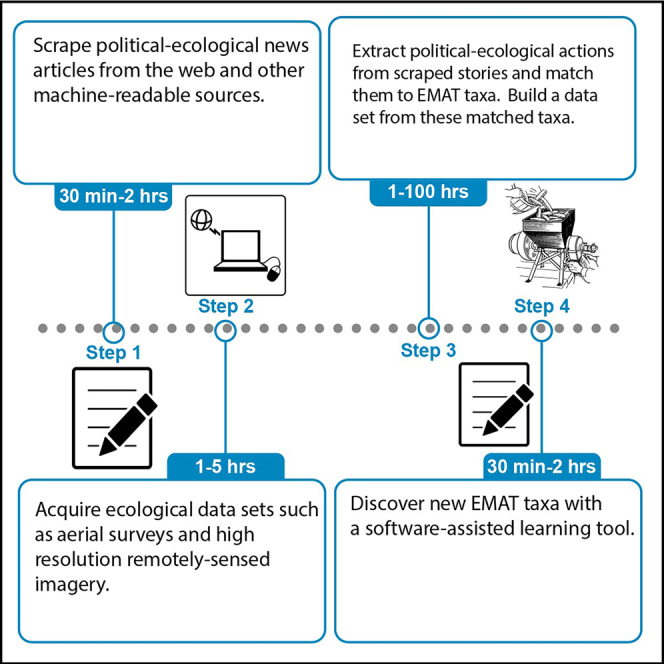

## Before you begin

This protocol has been used to build databases for the white rhinoceros (*Ceratotherium simum*) ecosystem in South Africa,^1^ and the ecosystem that hosts cheetah (*Acinonyx jubatus*) in East Africa.^2^ These studies focused on human-wildlife interactions. The protocol can also be used to gather taxonomy-indexed data on ecosystems impacted by anthropogenic forces such as pollution or habitat encroachment.

In general, a *taxon* is one of the categories of a taxonomy. Because the EMAT categorizes actions, in what follows, an EMAT taxon is referred to as an *EMAT action*. Also, in database theory, an entry in a database such as an existing student in a school’s enrollment database is referred to as an *entity*. Therefore, a real-world action reported by a machine-readable source and extracted by this protocol as an exemplar of a particular EMAT action, is referred to as an *EMAT entity*.

The EMAT is an extension of a political actions taxonomy developed by Leng.^3^ Currently, the EMAT consists of 119 militaristic actions, 191 diplomatic actions, 198 economic actions, 92 ecosystem-directed anthropogenic actions, and 37 *ecological actions*. Each action is associated with a set of archetypal actors. Ecological actions are of various types including species abundance, habitat metrics such as vegetation index, wildlife disease outbreak events, events of wildlife-caused damage to crops, and events of wildlife attacks on humans.

Each EMAT action can be decomposed into at most, three sentence components: an *m-word verb*, a direct object phrase, and/or a prepositional phrase. Letting *m* be a positive integer, an *m*-word verb subsumes single-word verbs (either *regular* or *irregular*), and *multi-word verbs* (those that use more than one word to convey their meaning, e.g., “picked up”); also known as *phrasal verbs*.^4^

These decompositions are realized in the file, parsedematacts.dat wherein each EMAT action has been manually parsed into three *equivalence sets*: A set of semantically equivalent *m*-word verbs, a set of semantically equivalent direct object phrases, and a set of semantically equivalent prepositional phrases, respectively.

An EMAT database^2^ holds observations of these actions (EMAT entities) that have been gleaned from a variety of sources including machine-readable stories, pre-analyzed remotely sensed images, and published surveys of wildlife abundance. A *story* will refer to a machine-readable news article, social media post, government report, or a scientific report. The World Wide Web contains a massive number of such stories that are being added to daily. Many of these stories may be viewed for free.

A JAVA program written by the author, called **id** (for Influence Diagrams) executes several steps of this protocol. Hereafter, this program is referred to as the **id** *software package*.

## Key resources table

**Table:** 

REAGENT or RESOURCE	SOURCE	IDENTIFIER
**Software and algorithms**		

catstories.bat	This paper; Data S1	N/A
catstories.ps1	This paper; Data S1	N/A
downloadalertsvba.txt	This paper; Data S1	N/A
getalerts.bat	This paper; Data S1	N/A
getnews.bat	This paper; Data S1	N/A
getnews.ps1	This paper; Data S1	N/A
phrases.bat	This paper; Data S1	N/A
wsearch.bat	This paper; Data S1	N/A
emat.dfn	This paper; Data S1	N/A
parsedematacts.dat	This paper; Data S1	N/A
efgroups.dat	This paper; Data S1	N/A
efregions.dat	This paper; Data S1	N/A
∗.java	This paper; Data S1	N/A
compile.bat	This paper; Data S1	N/A
idalone.bat	This paper; Data S1	N/A
extract.id	This paper; Data S1	N/A
ef9-13.txt	This paper; Data S1	N/A
ef9-13ents.dat	This paper; Data S1	N/A
taxonomyoverview.pdf	This paper; Data S1	N/A

**Other**		

JAVA Developer’s Kit (JDK)	Oracle Corp.	https://oracle.com/java/technologies/downloads
cat.exe	GNU	https://gnu.org/software/coreutils/
grep.exe	GNU	https://gnu.org/software/grep/

## Materials and equipment

Key resources for this protocol include utilities for web scraping, EMAT entity extraction, and learning new EMAT actions. Batch files have a .bat extension; PowerShell programs, a .ps1 extension; and Outlook macros, a .txt extension. Story files also have a .txt extension.

All files listed under **Software and algorithms** in the key resources table are in Supplementary material Data S1.

The scripts and JAVA program listed in the key resources table will run on a Windows 11 computer. The **id** software package can extract entities at scale by analyzing stories in parallel. This is accomplished by taking advantage of the *embarrassingly parallel search* (see Malapert et al.^5^) characteristic of the entity extraction step: The extraction of entities from one story is independent of the extraction of entities from some other story. Therefore, a cluster computer having *m* compute nodes, each with at least four processors can deliver an *m*-times speed up in the processing time of *n* stories when n≫m.

## Step-by-step method details

### Acquire stories and ecological data from machine-readable sources

**Timing: 4 h**

**Timing: 30 min (for step 1)**

**Timing: 1 h per 50 Alert emails (for step 2)**

**Timing: 1 h per 50 Alert emails (for step 3)**

**Timing: 30 min per 100 stories (for step 4)**

**Timing: 1 h (for step 5)**

Scrape stories from either existing machine-readable files or the World Wide Web utilizing a variety of program files (see the key resources table). Download all batch, PowerShell, and Outlook macro files listed in the key resources table to a single folder, e.g., C:*∖*pedatacq (for *political- ecological data acquisition*).

**Figure 1:**
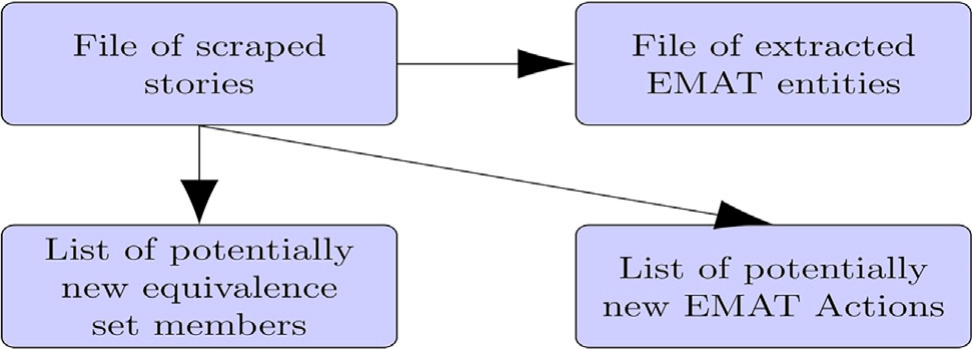
Expected outcomes from running this protocol The protocol begins by producing a file of stories scraped from machine-readable sources. The protocol then extracts actions from these stories that match actions in the Ecosystem Management Actions Taxonomy (EMAT). The protocol finishes by assisting humans to learn both, new phrases to add to a particular EMAT action’s equivalence sets; and entirely new EMAT actions.

1. Aggregate existing, separate story files. Produce a story file from a set of separate individual story files that is formatted for the EMAT extraction and learning steps, below.
  a. Use a file naming scheme that allows the collection of files to be referred to with *wildcard* notation.
    ***Note:*** For example, files named as story1.txt, story2.txt, ... may be collectively referred to as story∗.txt.
  b. Edit catstories.bat to specify the collection of files to be aggregated and to specify the aggregation file as follows.
    c:/pedatacq/catstories.ps1 *wildcard filenames append file name*
  c. Run catstories.bat to produce (a) the line “beginarticle 0” being inserted as line 1 in each file, and (b) all of these files being concatenated into one file.
2. Manually process Google Alerts. Produce a story file from a set of Google Alert emails that is formatted for the EMAT extraction and learning steps, below. This substep manually sets up a Google Alerts service, captures the ensuing Alerts emails, downloads all Alerts emails, and finally, downloads each Alert’s story.
  a. Create a (free) Google account.
  b. Create a Google Alerts service for a set of desired keyword phrases.
    i. To add or delete a Google News Alert, log into your Google account by accessing the link www.google.com/accounts.
      ***Note:*** Your username is your email address.
    ii. Click Dashboard (click Accounts and then click Alerts).
  c. Send these alerts to a folder in your mailbox as follows. In Outlook Web Access (OWA), click Settings and then Options. Or, click Settings, Show all Settings, and then Options.
    ***Note:*** Worst case, you may have to enter options as a search word to get to that item. Once there, click Inbox Rules.
    **CRITICAL:** Once a Google Alerts service has been started, it will autonomously and continually send Alert emails to the designated folder at the user’s email address. This can amount to 500 emails per month. Weekly monitoring of this folder can avoid email access failures due to the receipt of an excessive number of emails.
  d. The manual substep for downloading an OWA folder is as follows.
    i. Start a local Outlook session and login to the connected OWA. Let the local Outlook application finish updating.
    ii. If not present as a dotted line icon in the very top row (left) of the local Outlook application’s icons, add the Select All command to its Toolbar. Do this by selecting All Commands in the Choose Commands list, scrolling to the Select All command, and then clicking Add.
    iii. Get into the desired folder, e.g., googleeastaf and then click the Select All icon.
    iv. Click File and then Save As. Take the default (Text Only). Give a file- name with a .dat extension, e.g., allalerts.dat. Call this the *alerts file.*
      **CRITICAL:** Do not click more files on Exchange because this will cause the URL of each Alert’s story link to not be written to allalerts.dat.
  e. Open each alert email and click each story’s link and write it to a file as an HTML-only file type (“webpage, HTML only” format).
    ***Note:*** Use a filename of story*n*.htm where *n* is a number, e.g. story1.htm, story2.htm, ....
3. Substeps (a) through (e), above have been automated in the file getalerts.bat. Manually run this batch job by typing C:/pedatacq/getalerts at a Windows command prompt.
  **CRITICAL:** Only after starting this batch job, start a local version of Outlook.
  a. After this local version of Outlook has finished updating, do the following.
    i. Click Developer, Macros, and then downloadalertsvba.
    ii. Choose the folder to be downloaded, e.g., googleeastaf.
    iii. After the macro finishes, verify that this folder is now empty on both the local version of Outlook and on OWA.
    iv. Hit any key to continue the getalerts batch job.
  b. This batch file continues by scraping the stories off the web pointed to in the downloaded emails. The program does this by concatenating and cleaning these downloaded Alert files as follows.
    i. Concatenate all Google Alerts emails into one alerts file.
    ii. Clean this file by first, removing ˆ@ control characters via the device of converting the file from UTF-16 to ANSI. Second, replace the beginning and ending string added by Google and Outlook in every story URL with a space so that getnews.ps1 can successfully read and then connect to these URLs. These replacements need to be performed in the following order.
       Replace %3A with:
       Replace %2F with /
       Replace %3D with a space.
       Replace %26 with a space.
       Replace *<*https://nam02.safelinks.protection.outlook.com/?url= with a space.
       Replace https://www.google.com/ with a space.
  c. Download each story pointed to by a link in the alerts file by executing the following command.
    powershell c:/pedatacq/getnews.ps1 googlealerts
    *alerts filename append filename*
    The second argument to this command is the alerts file, and the third is the file to append the downloaded stories to.
4. Acquire stories from commercial news aggregators. Use a free commercial news aggregator to find stories. The PowerShell program getnews.ps1 (called from getalerts.bat) is reused from above to scrape stories from the commercial news aggregator, Newsapi.
  a. Setup a free account at www.newsapi.org.
  b. Schedule the program getnews.bat to run at a specified time and day every week with Task Scheduler, found at Windows Administrative Tools > Task Scheduler. Do this as follows.
    i. Edit this task by clicking Task Library and then clicking on the task.
    ii. Click Properties from the menu on the right of the screen. Then, click the characteristic of the task to be edited and then click Edit.
    iii. For this task’s Action, specify Run a Program and then enter the full path to the program getnews.bat, e.g., c:/pedatacq/getnews.bat.
    iv. Set the General options of the task to: runs only when the user is logged on.
5. Acquire ecological data from machine-readable sources.
  a. Acquire species abundance data as follows.
    i. Enter into a web search engine phrases that mention the species of interest and its general location.
      ***Note:*** For example, *East African cheetah* was used to acquire cheetah abundance data.^2^
    ii. Read the research articles that are returned from this search and manually extract abundance data given in each article. Otherwise, download the data sets from data repositories pointed to in these articles.
  b. Acquire satellite images of large mammals and/or large flora as follows.
    i. Identify the box of longitude and latitude coordinates of the area of interest.
    ii. Contact a commercial satellite imagery provider such as MAXAR (www.maxar.com) or Airbus Defence and Space (www.intelligence.airbus.com) and purchase images of this area taken at satellite fly-over dates that are closest to those desired.
    iii. Once purchased, download the desired images.
    iv. Create a one-sentence story for this data set.
      ***Note:*** From Haas^2^: “Entities in the data set reference table (table ecodatref in Figure 1) are observations on the *collect data* EMAT action and have seven attributes: source, species, type, country, region, startdate, and enddate. The source attribute is either observation, or model. The species attribute indicates the observed species, e.g., **cheetah, rhino,** or **cycad** (*Cycadophyta*). The type attribute can take on the values of abundance, presence/absence, capture-recapture, rainfall, NDVI, and landuse. For these latter three values, the species attribute is set to N/A. These data set references are preprocessed into one-sentence stories of the form ``group name collected type data on species in region, country during the period startdate to enddate.’’ group name is the group who collected data, e.g., **Kenya Wildlife Services, SANParks Scientific Services,** or **TerraServer.**

### Extract EMAT entities

**Timing: 1 h per 1,000 stories**

This step extracts EMAT entities from the story file.

6. Locate the story file that was created by substeps 1 through 4.
7. Run the **id** software package’s relation, parse stories() to extract sentences, *m*- word verbs, direct object phrases, prepositional phrases, and EMAT entities from the stories in this story file.
  ***Note:*** For example, the **id** software package’s input file, extract.id (see the key resources table) extracts actions from the story file, ef9-13.txt:
  report prepare data parse stories(2
   c:/polbio/stories/ef9-13.txt
   false false 1 efgroups.dat efregions.dat ef9-13sdb.dat ef9-13ents.dat).
  The parse stories() relation executes the following steps.
  a. Scan each story for the story’s source.
  b. Remove a pre-selected set of HTML tags from the story to produce a *tag-filtered story.*
  c. Form a text fragment of the story that consists of textual content sentences only.
    ***Note:*** A sentence contains textual content if (a) it contains at least three *common words* defined by the list *{the, a, of, is, by, to, be, from, and, have, in, that, on, with, as, at, inside}*; and (b) less than 80% of its words are *irrelevant* as defined by the list *{content, copyright, stylesheet, subscribe, subscription, login, header, sidebar, wrapper, label, navigation, class, column, http:, republish, div}*.
  d. Search each tag-filtered story for its date, groups, regions, and EMAT entities. In particular, extract EMAT entities with the *EMAT entity extraction algorithm.*
    ***Note:*** These four searches are performed simultaneously by running each search in its own, independent *thread.* A speed-up will accrue when the code is run on a stand-alone computer or cluster computer node that has at least four processors – one for each thread.
    ***Note:*** The EMAT entity extraction algorithm searches a sentence for *m*-word verbs that *partially match m*-word verbs that are members of an EMAT action’s *m*-word verb equivalence set. For example, say that some hypothetical story contains the sentence,
    Five poachers were arrested on June 10, 2019 and sentenced to prison on August 8, 2019.
    This sentence contains two 1-word verbs: *arrested*, and *sentenced*. Similar searches are executed to find direct object phrases, and prepositional phrases^6^ that partially match members of corresponding equivalence sets. Parsing is accomplished with a modified version of the shallow parsing algorithm of Daelemans et al.^7^
    The EMAT entity extraction algorithm is as follows.
    i. Using the *phrase similarity* sub-algorithm, search a sentence for an *m*-word verb that best matches entries in *m*-word verb equivalence sets. Declare a match if a pair’s similarity (*SIM*) is greater than 0.95. Let this best-matching EMAT action be a member of action cluster *j.* Phrases are allowed to appear in any order within a sentence.
      ***Note:*** In the following phrase similarity subalgorithm, an *n-gram* is a sub-sequence of *n* words in a natural language phrase. One definition of the degree of similarity between two phrases is the *Phrasal Overlap Measure* of Ponzetto and Strube.^8^ Haas^9^ describes a modified version of this measure. In-turn, an improved version of that measure is,
      $$SIM\left(ph_{1},ph_{2}\right)\equiv \left(N/\left|ph_{2}\right|\right)\tanh\left[\frac{1}{s}\sum_{n=1}^{N}m_{n}n^{2}\right]$$
      where the number of words in phrase 1 is $N=\left|ph_{1}\right|\leq \left|ph_{2}\right|$; s is the number of times *n*-gram pairs are formed by starting at the same location in each phrase; and $m_{n}$ is the number of *n*-grams that are common to the two phrases.
      ***Note:*** A pair of *n*-grams is declared to be common if 1.0 minus the *Levenshtein distance*^10^^,^^11^ between the two is greater than 0.99. If $\left|ph_{1}\right|=\left|ph_{2}\right|=1$, $SIM\left(ph_{1},ph_{2}\right)$ is 1.0 minus the Levenshtein distance between the two.
    ii. If no matches are found, return null.
    iii. Initialize the variables *SIMD* and *SIMP* to 0.0.
    iv. Search the sentence for a direct object phrase that matches an entry in direct object phrase equivalence sets of EMAT actions in action cluster j. Declare a match if SIM>0.95. Store the matched EMAT action with the highest similarity score in *actionD* and store its similarity value in *SIMD.*
    v. Search the sentence for a prepositional phrase that matches an equivalence set prepositional phrase of one of the actions in action cluster j. Declare a match if SIM>0.95. Store the matched EMAT action with the highest similarity score in *actionP* and store its similarity value in *SIMP.*
    vi. If *actionD* and *actionP* are both null, return null.
    vii. If SIMP>0.8 return *actionP.*
    viii. If SIMD>SIMP, return *actionD.* Otherwise, return null.
  e. In the final list of extracted entities, check each entity for possible duplicates of it. Do this as follows.
    i. When/if a duplicate is found, remove it.
    ii. After a duplicate is found, search the list again for additional duplicates.
    iii. Move to the next entity only when no duplicates are found for the current entity.
8. Examine the output file, parsefailed.dat for stories that the parsing algorithm failed on.
9. Rerun all story files after improvements to the EMAT entity extraction algorithm are made and/or associated lexicon files are updated.

### Learn new equivalence set members and EMAT actions

**Timing: 30 min**

EMAT actions and their equivalence sets do not define a static taxonomy but rather a dynamic one as both the language evolves and new interactions between humans and ecosystems emerge. This dynamic characteristic of the EMAT is operationalized with a software-assisted human learning algorithm that can identify either a new equivalence set member of an existing EMAT action or, more fundamentally, an entirely new EMAT action.

10. Create several strings of one to four words that are thought to describe in-part, an EMAT action.

***Note:*** For example, the EMAT action Elephants trample crops could also be described with the strings *elephants destroyed fields* or *smashed by elephants*.

11. Locate the story file that was created by substeps 1 through 4.
12. Run the file, phrases.bat with these strings on this story file. View the output file for common usage of these strings in stories contained in the story file. These instances will suggest *m*-word verbs, objects, and prepositional phrases to add to that EMAT action’s equivalence sets in parsedematacts.dat.
13. Detect those sentences in a set of stories that have a *SIMP* score between 0.7 and 0.8 – or where SIMD>SIMP. Create a list of these *{*sentence, EMAT action*}* pairs.

***Note:*** The EMAT extraction algorithm, above, uses a *SIMP* value of 0.8 or greater to extract an EMAT entity.

***Note:*** These scores compare phrases to existing EMAT actions. Therefore, there is always a particular EMAT action that is associated with a *SIMP* score or a *SIMD* score.

14. Examine each sentence in this list to determine if it is clearly describing an occurrence of any existing EMAT action. For such a sentence, add the *m*-word verb, direct object phrase, and prepositional phrase to the equivalence sets of the associated, existing EMAT action.

**CRITICAL:** When updating parsedematacts.dat, if there are highly relevant words that need to be detected for a particular EMAT action, keep short the prepositional phrase that contains the needed words. That way, the similarity measure will be large only if those words are present in the story’s sentence.

***Note:*** As an example of discovering new equivalence set members, the following sentence gives a high *SIMD* score for the EMAT action Sell a few rhino horns.

In 2014, Kenya enacted tough new laws that make ivory poaching and trafficking punishable by fines of 200,000 USD or even life in prison compared to the maximum fines of about 400 USD that were handed out previously.

Clearly, this sentence describes an entity of the EMAT action: *tighten wildlife agreement or laws*. Hence, the 1-word verb *enacted* should be added to this action’s *m*-word verb equivalence set, and the phrase *tough new laws that make ivory poaching and trafficking punishable* should be added to the action’s direct object phrase equivalence set.

15. Examine the sentence for an ecosystem-relevant militaristic, diplomatic, economic, ecosystem-directed, or ecological action. If a new EMAT action is being described, use the sentence’s *m*-word verb, direct object phrase, and (if present) the sentence’s prepositional phrase as the initial members of this new EMAT action’s three equivalence sets, respectively.

***Note:*** As an example of discovering a new EMAT action, the following sentence from the file ef15-16.txt (see Table 1) produces a high *SIMP* score for the EMAT action Sell a few rhino horns but is clearly not describing that or any other existing EMAT action.

TenBoma is a communications based initiative that uses modern technology and sophisticated data analysis to allow law enforcement agencies to predict poaching plots in advance and thwart the incidents.

**Table 1:** Story, database, and entities history files in the author’s collection

Story file size (bytes)	Story file	Story database file	Actions history file
**Management of cheetah (*Acinonyx jubatus*) in East Africa**			

38,675,592	ef9-13.txt	ef9-13sdb.dat	ef9-13acts.dat
291,897,345	ef13-14.txt	ef13-14sdb.dat	ef13-14acts.dat
190,719,056	ef14-15.txt	ef14-15sdb.dat	ef14-15acts.dat
190,276,554	ef15-16.txt	ef15-16sdb.dat	ef15-16acts.dat
120,033,188	ef16-19.txt	ef16-19sdb.dat	ef16-19acts.dat
123,923,566	ef19.txt	ef19sdb.dat	ef19acts.dat
461,581,001	ef19-21.txt	ef19-21sdb.dat	ef19-21acts.dat
763,133,494	alerts21.txt	knp21asdb.dat	knp21aacts.dat
50,116,127	alerts22.txt	knp22asdb.dat	knp22aacts.dat

**Management of white rhinos (*Ceratotherium simum*) in South Africa**			

94,489,580	knp13-20.txt	knp13-20sdb.dat	knp13-20acts.dat
65,718,985	newsapi21.txt	knp21bsdb.dat	knp21bacts.dat
189,123,060	newsapi22.txt	knp22bsdb.dat	knp22bacts.dat
93,591,176	newsapi23.txt	knp23sdb.dat	knp23acts.dat

The file name pattern ∗sdb.dat is a story database file (one set of entities per story), and ∗acts.dat is an entities history file.

Instead, this sentence appears to be describing a new ecosystem management action that is about the introduction of a new technology to combat wildlife trafficking. Hence, the action and associated equivalence set members as shown in Table 2 should be added to the EMAT definition file, emat.dfn, and parsedematacts.dat, respectively.

**Table 2:** A new EMAT action along with its initial equivalence set members

Database entity	Value of its *phrase* attribute
Action	Develop new technology to combat wildlife trafficking
*m*-Word verb	*Uses*
Direct object phrase	*Modern technology and sophisticated data analysis*
Prepositional phrase	*To predict poaching plots*

## Expected outcomes

The protocol’s story-scraping step produces a file of stories scraped from machine-readable sources (Figure 1). These stories are formatted for further processing by the protocol’s EMAT entity extraction step. This step in-turn, produces a file of EMAT entities that are tagged by date, location, reporting source, actor, and target. The protocol’s learning step produces a list of potentially new equivalence set members for existing EMAT actions, and a list of potentially new EMAT actions along with their initial equivalence sets.

## Quantification and statistical analysis

### Assessing extraction accuracy

Haas^9^ discusses algorithms designed to extract EMAT entities from media:

The algorithm’s accuracy can be assessed by comparing the actions extracted from a random sample of stories to those extracted by a human reading the same set of stories. Using the set of human-extracted actions as the benchmark, the algorithm can make two types of errors: failing to extract an action in a story; and extracting an action that does not exist in the story, referred to here as a *spurious action*.

Let $n_{true}$ be the number of human-extracted actions from a story file. Let $r_{correct}$ be the fraction of the $n_{true}$ entities that were extracted by the algorithm. When this value is 1.0, the extraction algorithm is performing as well as a human. Values less than about 70% indicate improvements need to be made to the algorithm and/or phrases need to be added to the equivalence sets of those actions that are being missed by the algorithm. Let $r_{spurious}$ be the ratio of the number of spurious actions to $n_{true}$. Ideally, this value would be zero. Values larger than 40% indicate the equivalence set members of spuriously-matched actions are too short and/or are ambiguously-worded so as to match with many story text strings that actually carry different meanings.

When the **id** relation, parse stories() executes, an *extraction assessment* file is written that contains ten story texts along with those entities extracted by the algorithm. These texts have been uniformly sampled from the story file. The values of $r_{correct}$ and $r_{spurious}$ can be computed by manually reading this extraction assessment file and noting the number of true actions; the number of extracted actions that match true actions; and the number of extracted actions that are spurious. This assessment procedure is as follows.

1. Run parse stories() on a story file.
2. Open the extraction assessment file, check.dat in a text file viewer.
3. Find the total number of lines in this file, *m* and then read a story every m/10 lines.
4. Record $n_{true}$, $n_{correct}$, and $n_{spurious}$ for each of these *m* stories.
5. Simultaneously, record frequent spurious actions and actions that are not detected in order to improve the extraction algorithm by editing parsedematacts.dat accordingly.
6. From these values, compute an estimate of the extraction algorithm’s accuracy for that story file.

**Example,**

Extraction accuracy statistics for the story file, ef9-13.txt are $n_{true}=19$, $r_{correct}=0.74$, and $r_{spurious}=0.37$.

## Limitations

This protocol uses scraped stories as data for an EMAT entity extraction algorithm. Although web scraping is legal in the United States of America (see www.techcrunch.com/2022/04/18/web-scraping-legal-court/), publishing scraped story files would require copyright permissions for potentially hundreds of stories. Doing so may be cost prohibitive. Other limitations of this protocol are as follows. Any story that is not machine-readable will need to be scanned or otherwise converted to a machine-readable form. The protocol’s story-scraping step depends on the availability of external web crawlers, specifically, Google Alerts and Newsapi. The EMAT entity extraction algorithm uses a shallow parsing algorithm. Superior parsing algorithms may exist.

## Troubleshooting

### Problem 1

It may be that the local Outlook application decides that many of the Google Alerts emails are junk.

### Potential solution

When a local version of Outlook is started, Outlook may move these to the Junk folder on both the local and OWA applications. Once updating has finished, follow substeps (a) through (e) in step 2 for downloading Alerts emails from the Junk folder after you have performed a search of the Junk folder for “Google Alerts” emails.

### Problem 2

The **id** software package may run out of memory.

### Potential solution

If the Insufficient Memory error occurs and the **id** software package terminates abnormally, increase the values of the maximum memory options given to the JAVA machine to at least 8 GB by editing the file idalone.bat to: -Xms8024m, -Xmx8024m, and --max-mem-size = 8024 Mb.

### Problem 3

The **id** software package may appear to hang while processing a story file.

### Potential solution

The operating system may be thrashing, i.e., spending most of its processing time on transferring virtual memory in and out of low speed storage. In this case, first, terminate the job, and then second, increase the computer’s RAM before rerunning the **id** software package.

### Problem 4

A particular EMAT action may frequently appear as a spurious action.

### Potential solution

Edit parsedematacts.dat so that this action’s equivalence set members consist of more than four words each.

### Problem 5

parse stories() may fail to detect story dates in stories that are scraped from a new source.

### Potential solution

First, manually examine the HTML code of one of these problematic stories and find the story’s date and the particular HTML tag that contains that date. Second, add code to Storydate.java to read this new format. Finally, recompile Storydate.java.

## Resource availability

### Lead contact

Timothy C. Haas, Sheldon B. Lubar College of Business, University of Wisconsin at Milwaukee, 3202 N. Maryland Ave., Milwaukee, Wisconsin, 53201, United States of America, haas@uwm.edu.

### Technical contact

Timothy C. Haas, Sheldon B. Lubar College of Business, University of Wisconsin at Milwaukee, 3202 N. Maryland Ave., Milwaukee, Wisconsin, 53201, United States of America (haas@uwm.edu).

### Materials availability

In this protocol, story files are (scraped) materials. The story files used in examples above contain copyrighted material from many different news organizations. Acquiring publication permissions for all of these stories was cost and time prohibitive for this author. For this reason, no story files have been deposited in a publicly accessible data repository.

See Table 1 for a list of the author’s collection of story files scraped over the years 2009 to 2023. A file with a .txt extension is a story file that has been scraped off the web with minimal attempts at removing HTML fluff.

The files generated by getnews.bat are named newsapi202ymmdd.txt where for example, newsapi20230528.txt is a story file created on May 28, 2023. Let “202x” be the most recent full year, e.g., 2023. Each year’s Newsapi files have been concatenated with the following two commands.

• cat newsapi202x∗.txt > dum.txt.
• move dum.txt newsapi202x.txt.

getnews.bat is currently written to generate a story file of stories about rhino poaching only. This can be changed to other species and locations by editing getnews.bat accordingly.

### Data and code availability

All program files listed under Software and algorithms in the key resources table have been written by the author and are in Supplementary materials Data S1**.**

Database and actions history files are generated when the **id** relation, parse stories() is run on a story file. As an example, the **id** software package input file, extract.id runs the story file, ef9-13.txt and writes the output file, ef9-13ents.dat. These three files are included in Data S1. This story file contains articles from 2009 through 2013.

## Supplemental information

Data S1. Java scripts for implementation of EMAT, related to the key resources table
