# Supplementary material for: Protocol to discover machine-readable entities of the ecosystem management actions taxonomy
Source: STAR Protoc. 2024 Jun 13;5(2):103125. doi: 10.1016/j.xpro.2024.103125 (PMC11225903; doi:10.1016/j.xpro.2024.103125)
Supplement: Data S1. Java scripts for implementation of EMAT, related to the key resources table [file mmc1.zip › keyresources/taxonomyoverview.pdf]

Science, Taxonomies, and Large Language Models (LLMs)

Timothy C. Haas
Lubar College of Business
University of Wisconsin at Milwaukee
haas@uwm.edu

Taxonomies and the development of conservation science

A taxonomy like the EMAT, can be used to map a potentially large number of possible political-ecological actions to a finite number of actions that cover most things that humans can do to an ecosystem along with the ways an ecosystem can respond to those actions. Having a finite set of possible actions allows models of the interactions between humans and ecosystems to be specified in terms of a set of archetypal political actions taken by ecosystem-affecting actors, and archetypal ecological actions taken by the ecosystem in response to those political actions. This finite set of possible political-ecological actions does not grow with the size of a political-ecological actions data set (sample size). By adopting the sampling paradigm of making observations on a finite number of variables, political-ecological models fitted to such data can themsleves be expressed through a finite set of variables rather than through an exploding stream of observations. This makes these models parsimonious and hence, mappable to archetypal-based theoretical constructs in sociology, political science, and ecology.

Because EMAT actions form the bedrock of political-ecological theory construction, they are the fundamental variables of the emerging science of conservation. Defining a finite set of fundamental variables is Stage I in the development of a new science[1]. With the EMAT and this protocol, collecting data for purposes of developing theory and testing hypotheses in conservation science can proceed as in any other science. For instance, in medical science, there are a finite number of fundamental variables such as body temperature and blood pressure. To test hypotheses about the effect of Covid-19 on body temperature for example, observations on body temperature are collected from a sample

of humans who are Covid-19 positive.

Most EMAT actions are nominally-valued variables, e.g. the presence or absence of a rhino poaching event on a particular day and in a particular region. In medical science, a similar variable would be whether a particular human's TP53 gene is normal or mutated (either through inheritance or spontaneously)[2]. Haas[3] discusses the EMAT's role in the stages involved in the development of a science:

> Shneider (2009) describes four stages that a new science such as conservation, goes through as it develops. These are (I) the identification of its fundamental objects, phenomena, and language to describe its subject matter; (II) creation of tools for studying these objects and phenomena; (III) discovery of mechanisms that predict observed phenomena; and (IV) broadcast and maintenance of this predictive knowledge. This article delivers a taxonomically-based relational database of political-ecological physical actions, verbal actions, and data. It further develops the concept of an episode of political-ecological actions, and gives a tool for determining if such an episode is causal. These two breakthroughs: an EMAT database and attendant episode analysis give, for the first time, a language to conservation science. This language enables researchers to identify what data needs to be collected, and what a theory of political-ecological systems should be able to explain. In particular, this theory should offer data-verified causal mechanisms that produce the observed, coupled actions of political actors and ecosystem members. This operational triad of objects, phenomena, and language supports the convergence of conservation theories. Therefore, this article makes a fundamental contribution to conservation science because it completes stage I of a developing science through its EMAT database, and begins stage II through its introduction of episode analysis.

Table 1 contains a few of the more frequently encountered EMAT actions. Each EMAT action is associated with a set of archetypal actors. For example, the action `Complete electrified wildlife control fence` can be executed by either an environmental protection agency (EPA) or a non-governmental organization (NGO). Here, "EPA" is a generic

Category	Subcategory	Action	ID	Archetypal Actor $\rightarrow$ Target
	Military	`Arrest some poaching suspects`	MM0015	B $\rightarrow$ H
	Diplomatic	`Petition to stop wildlife-caused crop destruction`	D12719X3	F $\rightarrow$ A
Political	Economic	`Complete electric wildlife-control fence`	E23719X3	E $\rightarrow$ H
	Ecosystem directed	`Translocate animals`	C0008	B $\rightarrow$ K
	Ecosystem directed	`Poach some elephants`	CED15	F $\rightarrow$ K
Ecosystem		`Elephants trample crops`	Z006	K $\rightarrow$ F

Table 1: Frequently encountered EMAT actions. Archetypal actors and targets are denoted as: A = president, B = EPA, E = EPA or NGO, F = rural resident, H = rural resident or pastoralist, and K = ecosystem. Archetypal actors and targets are based on encounters with actual stories. The archetypal groups are used only when the EMAT entity extraction algorithm fails to find both an actor and a target in a story.

moniker for any governmental agency charged with protecting wildlife and/or the environment. Each EMAT action is implicitly associated with a particular scale of influence. For example, the scale of the EMAT action, `Poach for food` is regional, whereas the scale of the EMAT action `Strengthen wildlife protection laws` is national.

A taxonomy such as the EMAT is an ontology that represents only hierarchical relationships among the ontology's taxa[4]. A foundational paper on the *Semantic Web*[5] describes a taxonomy as a *simple ontology*[6,7]. Taxonomies are also known as *hierarchical ontologies*[8].

For *m*-word verb equivalence sets, if an EMAT action's *m*-word verb equivalence set contains a 1-word regular verb, then all conjugated forms of that verb are also included in the set. Such regular verbs are added to the file `parsedematacts.dat` in their past-tense form. Doing so supports the following verb conjugation algorithm employed in the **id** software package.

> When a 1-word verb ending in "ed" is read by **id** from the equivalence set file, all conjugated forms of that verb are immediately added to the *m*-word verb equivalence set for that action. For example, when the 1-word verb, *poached* is read, the 1-word verbs *poach*, *poaching*, and *poaches* are added to the equivalence set.

Phrasal verbs can be partially conjugated by their 1-word verb substitutions, if any[9]. Initial entries in an EMAT action's equivalence sets are phrases from stories that report unambiguous instances of the associated EMAT action.

Using an LLM to learn new EMAT actions

An LLM is a *next-token predictor* that takes the form of a multi-layer neural network that has been trained on a large, observational collection of texts[10]. The potential of LLMs to extract political-ecological actions with or without reference to a taxonomy, has not been explored. One potential use of an LLM is as follows. Enter into an LLM, the phrase "How do humans interact with wildlife?" The returned statements might contain new EMAT actions because the LLM has been trained on such a large corpus. A human trained in both political science and ecology would decide which of those actions to add to the EMAT. It is not clear, however, if LLMs should be used to structure an ontology of political-ecological actions because at their current level of development, there is no basis for believing that LLMs can reason and plan[11].

References

1. Shneider, A. M. (2009). Four stages of a scientific discipline; four types of scientist. Trends Biochem Sci. May, 34(5), 217-23. 10.1016/j.tibs.2009.02.002.

2. Hu, J., Cao, J., Topatana, W., Juengpanich, S., Li, S., Zhang, B., Shen, J., Cai, L., Cai, X., and Chen, M. (2021). Targeting mutant p53 for cancer therapy: Direct and indirect strategies. Journal of Hematology and Oncology, 14, Article number 157. 10.1186/s13045-021-01169-0.

3. Haas, T. C. (2021). The first political-ecological database and its use in episode analysis. Frontiers in Conservation Science, section: Planning and Decision-Making in Human-Wildlife Conflict and Coexistence, 2, 707088. 10.3389/fcosc.2021.707088. https://www.frontiersin.org/article/10.3389/fcosc.2021.707088.

4. American Society for Indexing (2018). Taxonomies & controlled vocabularies special interest group. www.taxonomies-sig.org/about.htm.

5. Yu, L. (2015). A developer's guide to the semantic web. 2nd ed. (Springer), ISBN: 978-3662437957.

6. McGuinness, D. L. (2005). Ontologies come of age. In Spinning the Semantic Web: Bringing the World Wide Web to its Full Potential, D. Fensel, J. Hendler, H. Lieberman, and W. Wahlster, eds. (The MIT Press), pp. 171-194, ISBN: 978-0262562126.

7. van Rees, R. (2003). Clarity in the usage of the terms ontology, taxonomy and classification. In CIB W78's 20th International Conference on Construction IT, Construction IT Bridging the Distance, R. Amor, ed. (Waiheke Island, New Zealand, 23-25 April), http://itc.scix.net/cgi-bin/works/Show?w78-2003-432.

8. Khan, S., and Safyan, M. (2014). Semantic matching in hierarchical ontologies. Journal of King Saud University - Computer and Information Science, 26(3), 247-257. 10.1016/j.jksuci.2014.03.010.

9. Bryson, S. (2022). List of 47 phrasal verbs and their one-word substitutions. Blog. https://www.scribbr.com/academic-writing/phrasal-verb-alternatives/.

10. Shanahan, M. (2024). Talking about large language models. Communications of the ACM, 67(2), 68-79. 10.1145/3624724.

11. Kambhampati, S. (2023). Can LLMs really reason and plan? BLOG@CACM, https://cacm.org/blogs/blog-cacm/276268-can-llms-really-reason-and-plan/fulltext.
